# Menstrual irregularity and its associated factors among college students in Ethiopia, 2021

**DOI:** 10.3389/fgwh.2022.917643

**Published:** 2022-08-23

**Authors:** Yohannes Moges Mittiku, Haile Mekonen, Girma Wogie, Michael Amera Tizazu, Getu Engida Wake

**Affiliations:** Department of Midwifery, Asrat Weldeyes Health Science Campus, Debre Berhan University, Debre Birhan, Ethiopia

**Keywords:** age of menarche, body mass index, college students, menstrual irregularity, perceived stress

## Abstract

**Background:**

Menstrual irregularity can occur at any age, but it is most common among women under the age of 23 years. Menstrual irregularity is a foremost gynecological problem and a cause of anxiety to students and those close to them. These students experience monthly absenteeism, premenstrual symptoms, and a lack of concentration due to menstrual problems, all of which interfere with their education. Therefore, this study aimed to assess the magnitude of menstrual irregularity and associated factors among college students in Debre Berhan Town, North Shewa, Amhara Regional State, Ethiopia, in 2021.

**Methods:**

An institution-based cross-sectional study was conducted from June to July 2021 in Debre Berhan town. Data was collected using self-administered questionnaires in 420 eligible female college students by systematic random sampling technique. Weight and height were measured and Body Mass Index (BMI) was calculated after data collection. Each questionnaire was checked for completeness, cleaned, coded, entered into EPI-DATA, and then transported to SPSS software. Bi-variable and multivariable logistic regression analyses were employed to determine the association of each independent variable with the dependent variable. *P* ≤ 0.05 were used to declare association and select predictors.

**Results:**

In the current study, 395 students participated with a response rate of 93.6%. Of all the total respondents, the magnitude of menstrual cycle irregularity was 33.4% (95% CI 28.6–38.2). Age < 20 years old [AOR = 3.88, 95% CI (1.25–12.18)], age of menarche ≤ 12 years [AOR = 4, 95% CI (1.18–13.9), sleeping hours ≤ 5 h [AOR= 2.26, 95% CI (1.04–4.93)], perceived stress [AOR = 2, 95% CI (1.53–3.23)] and being overweight [AOR = 2, 95% CI (1.13–3.23) were the variables significantly associated with the magnitude of menstrual irregularity.

**Conclusion and recommendation:**

This study shows that more than one-third of the college students in Debre Berhan town have experienced menstrual irregularity. Being less than 20 years old, having a history of early menarche, being overweight, and perceived stress were a variable significantly associated with menstrual irregularity. To control menstrual irregularity, girls should control their weight and lead a healthy lifestyle, including getting adequate sleep which could be aided by training on time management.

## Introduction

The menstrual cycle is the monthly set of changes that a woman's body undergoes in preparation for pregnancy. Hormones cause the lining of the uterus to become thicker with extra blood and tissue. The uterine lining sheds through the vaginal opening if ovulation occurs and the egg is not fertilized. The release of blood and tissue from the lining of the uterus is a menstrual period (1, 2).

Normal menstruation can last from 2 to 7 days and can happen every 21–35 days (1). However, 14–25 percent of women have irregular menstrual cycles, which means their periods are heavier or lighter than usual, longer than 35 days or shorter than 21 days, or they have other issues, such as abdominal cramps (3). In addition, bleeding or spotting in between periods, bleeding or spotting after sex, menstrual cycle length varying by more than 7–9 days, and/or not having a period for 3–6 months are menstrual irregularities (1, 3, 4).

At any age, menstrual irregularity can occur. However, it is usual for a woman's periods to be irregular at particular times in her life. When a girl initially starts having periods, they may not come on a regular basis (5). The anovulatory cycle, which is linked to the immaturity of the hypothalamic-pituitary-ovarian axis, is the most common cause of abnormal uterine bleeding (AUB) in adolescents during the first 19 months of menstruation. The common cause of AUB is a miscarriage, ectopic pregnancy, infection, hormonal contraception, stress, bleeding disorders, and endocrine disorders (6). Menstrual problems and AUB are two of the most common gynaecologic issues among teenagers (7).

Women under the age of 23 were the most likely to have menstrual irregularity (5). Menstrual irregularity was reported to be widespread in 35.7 and 64.2 percent of women in India and Nepal, respectively, according to several studies (8, 9). In addition, the prevalence of menstrual irregularity in Sudan was 55 percent (10). Furthermore, menstrual irregularity was reported to be prevalent in Ethiopia at 26.5–32.6 percent. Lack of adequate sleep, alcohol intake, stress, Anemia, hereditary factors, and being underweight are major contributing factors to menstrual irregularity (11–13).

Female students face many problems that challenge their quality of life and academic performance. Of the problems that affect the quality of life of female students, menstrual cycle irregularity is a foremost gynecological problem and a cause of anxiety to students and those close to them (14). It affects the different day-to-day events of students (15, 16). Different studies revealed that menstrual irregularities can also affect issues later on in life, such as osteoporosis, infertility, future diabetes mellitus, and cardiovascular disease (14, 17, 18).

College students have reported menstrual discomfort, feelings of guilt and sadness, and difficulty containing menses as bad menstrual experiences. These factors adversely affect their education through absenteeism, reduced engagement, and poor academic performance. Menstruation, on the other hand, can be a pleasant experience for some students, and their capacity to adjust to dysmenorrhea's problems indicates their perseverance and ingenuity. Monthly absenteeism, premenstrual symptoms, and lack of concentration cause issues in studies (19). As a result, this study aimed to assess the magnitude of menstrual irregularity using the International Federation of Gynecology and Obstetrics menstrual guidelines parameters for normal and abnormal uterine bleeding 2018's standard of menstrual irregularity definition (20).

## Methods and materials

### Study design, settings, and participants

An institutional-based cross-sectional study design was conducted at colleges in the Debre Berhan town from June to July 2021. Debre Berhan Town is the capital of the North Shewa Zone in the Amhara regional state, which is 130 km away from the capital of Ethiopia, Addis Ababa. There were three public colleges in the town and, during the physical year, the total population of the study area was 2,755.

### Population

All regular college students in Debre Berhan town during the study period were a study population. Students who were pregnant, had delivered, and were breastfeeding during the study year, had a history of menstrual irregularity therapy, or were using hormonal contraceptives throughout the data-collecting period were excluded from the study.

### Sample size and sampling procedures

**Figure 1:**
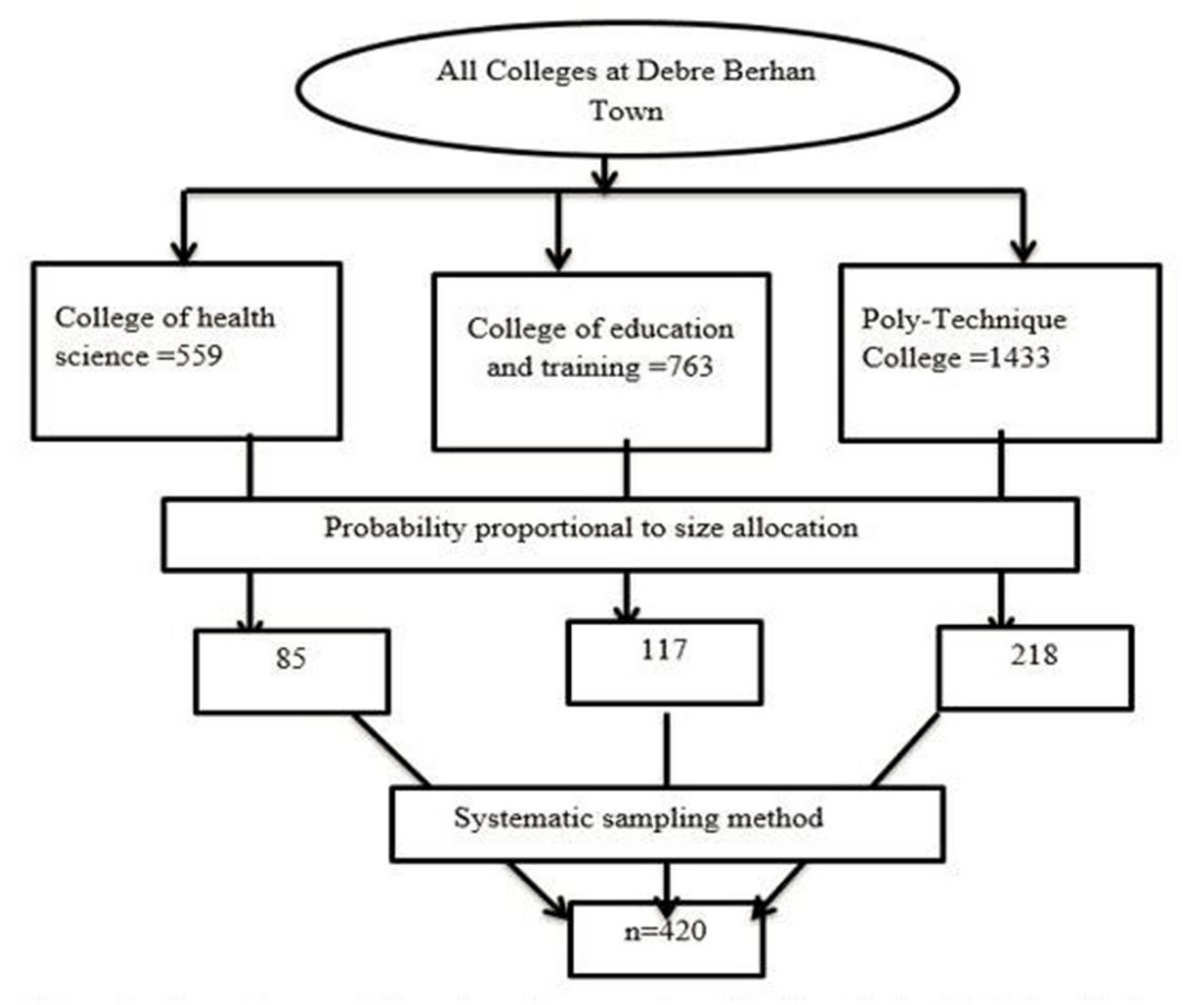
Schematic presentation of sampling procedure of college students in Debre Berhan town, 2021.

The sample size was calculated using a formula for estimating a single population proportion with a 95 percent confidence interval, a 5% margin of error, and the prevalence of menstrual irregularity as 50% and a 10% no-response rate. The total number of people in the sample was determined to be 422. All colleges in Debre Berhan Town were included in the study. The sampling allocation was also made based on the number of students in each college. For this study, a systematic random sampling method was used. Of all colleges, there were 2,755 (*N*) female students. The sample size was 420 (*n*); therefore, the skip interval was calculated as *K* = *N*/*n* where *K*, which is skipped interval, was seven. The starting point was chosen at random, and then everyone that corresponded to the skip interval was chosen starting from that point (Figure 1).

### Data collection procedures

The data was collected using a pre-tested self-administered questionnaire. Anthropometric measurements were performed to calculate the body mass index (BMI) of the participants. The height of participants was measured in meters and weight was recorded close to 100 g (least count of electronic weighing scale = 100 g). After analyzing several works of literature, the questionnaire was first written in English, then translated into Amharic to ensure that it was appropriate for female students, and then back to English to ensure consistency by a third party. To meet the study's aims, the questionnaire was built based on the variables. Sociodemographic information, menstrual-related questions, lifestyle and behavioral questions, medical history inquiries, and anthropometric measurements are all included in the questionnaire (height and weight).

### Data quality control and analysis

Every questionnaire was checked for completeness and consistency. As data collectors and supervisors, three diploma-trained midwives and one BSc-trained midwife were hired. The principal investigator provided data collectors and supervisors with training to address the study's relevance, objectives, and confidentiality concerns. Epi-Data was used to enter, edit, and clean data before being exported to SPSS version 21 for analysis. Frequencies were also subjected to descriptive statistics. Then, one by one, bivariate logistic regression was run between the dependent and independent variables. Variables with *P* < 0.25 in the bi-variable analysis were entered into a multivariable logistic regression to control the possible effect of confounders. Crude and adjusted odds ratios with h 95% confidence interval were computed. Finally, variables at *p* < 0.05 were considered statistically significant.

### Outcome measurement

The International Federation of Gynecology and Obstetrics (FIGO) 2018 criteria for menstrual irregularity definition have been used to establish if the menstrual cycle is regular or irregular. As a result, in the current investigation, a regular menstrual cycle was defined as having a frequency of 24–38 days, a duration of bleeding of less than or equal to 8 days, a cycle-to-cycle variance of fewer than 10 days during the previous year, and a normal individual assessment of the quantity (20). Menstrual irregularity, on the other hand, refers to anything that occurs outside of the normal menstrual cycle.

### Operational definition

#### Body mass index (BMI)

Body mass index (BMI) is defined is a measure for indicating nutritional status in adults. It is defined as a person's weight in kilograms divided by the square of the person's height in meters (kg/m^2^). Based on the calculated BMI, the study participants were classified as underweight (BMI < 18.5), normal weight (BMI 18.5–24.9), overweight (BMI 25–29.9), and obese (BMI ≥ 30) (21).

#### Perceived stress scale (PSS) assessed perceived stress

The PSS is a well-known stress assessment tool; it is a 10-item multiple-choice self-report psychological questionnaire used to assess stress perception. Each response was given a score ranging from 0 to 4. PSS is calculated by adding all scale items together. The total score ranges 0.0–21.0 [mean = 13.7 (± 6.6)]. The stress limit cut-off values were set at 15 (22).

#### Substance abuse

Substance abuse refers to the harmful or hazardous use of psychoactive substances, including alcohol and illicit drugs. One of the key impacts of illicit drug use on society is the negative health consequences experienced by its members. Drug use also puts a heavy financial burden on individuals, families, and society (23). Alcohol drinking was measured based on the frequency of alcohol drinking. Cigarette smoker was defined as someone who smokes cigarette either daily or occasionally.

#### Sleeping hour

Sleep duration was measured for just one sleep period or over the course of a 24-h day (24). We assessed the students' sleeping hours by asking, “How many hours do you sleep on average per day?”

## Result

### Socio-demographic characteristics

Three hundred ninety-five female students participated with a respondent rate of 93.6%. Most of the respondents (227 or 57.5%) were below 21 years old. The mean age of the study participants was 20.94 ± 2.10 years. The religion of most respondents 341 (90.9%) was orthodox. Regarding the respondents' ethnicity, 341 (86.3%), 39 (9.9%), and 15 (3.8%) were Amhara, Oromo, and Tigre, respectively. Of all participants, 86.3% were married. Concerning respondents' mothers' educational status, most of them 63.8% had no formal education, followed by primary education 26.8%. Regarding residence before college admission, 53.2% of them came from a rural area (Table 1).

**Table 1:** Socio-demographic characteristics of female college students, Debre Berhan, Ethiopia 2021 (*n* = 395).

**Variable**	**Categories**	**Frequency (*n*)**	**Percent (%)**
Age	≤ 20 years	227	57.5
	21–24 years	143	36.2
	≥25 years	25	6.3
Religion	Orthodox	359	90.9
	Catholic	20	5.1
	Others	16	4.0
Marital status	Single	341	86.3
	Married	54	13.7
Family marital status	Live together	335	84.8
	Divorced	51	12.9
	Windowed	9	2.3
Respondents' mothers' educational status	Had no formal education	252	63.8
	Primary school	106	26.8
	High school	22	5.6
	College and above	15	3.8
Respondents' monthly income (Ethiopian Birr)	≤ 500	258	65.3
	>500–1,000	98	24.8
	>1,000	39	9.9
Study year	First-year	35	8.9
	Second-year	139	35.2
	Third-year	221	55.9

Others, Muslim and protestant.

### Body mass index

**Figure 2:**
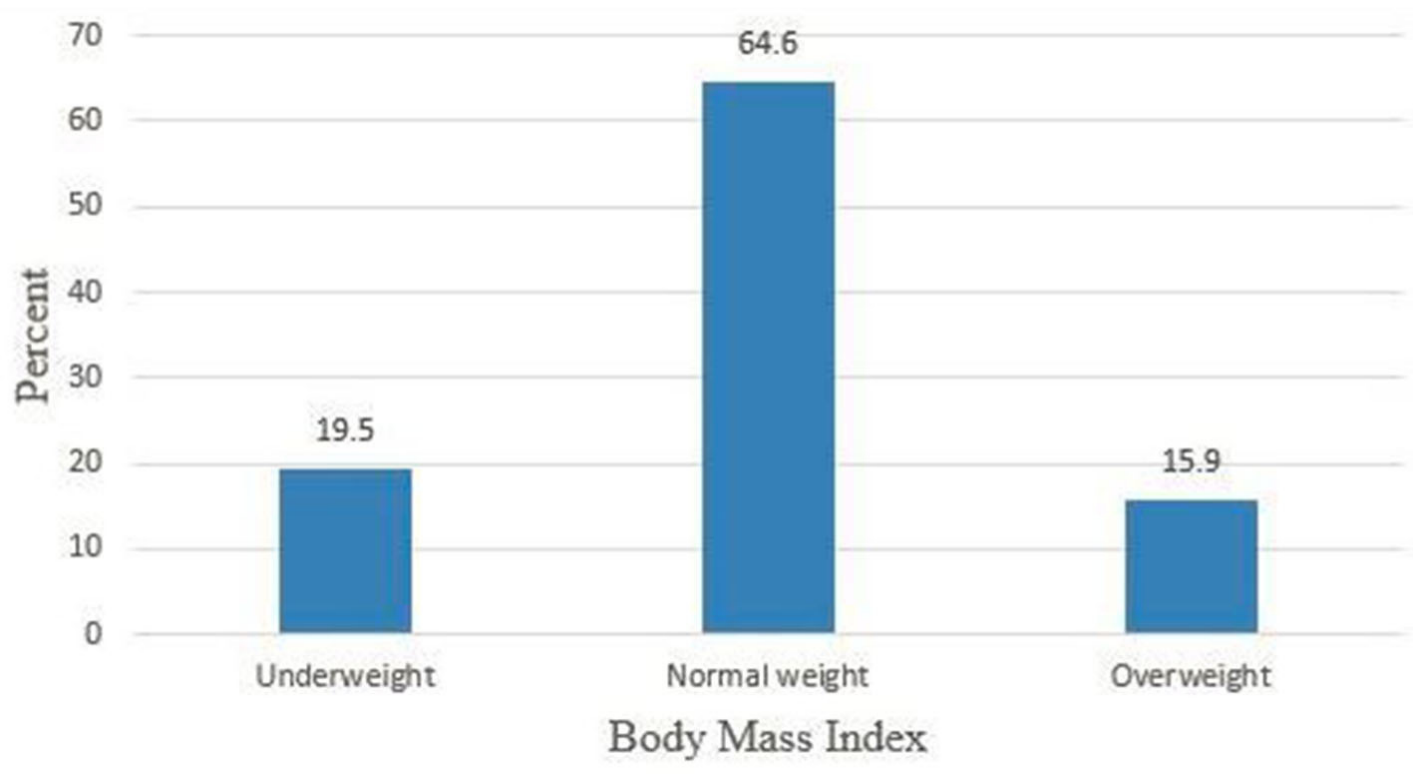
Body mass index of female college students, Debre Berhan, Ethiopia, 2021 (*n* = 395).

For all study respondents, body mass index was calculated and 77 (19.5%) respondents were underweight (Figure 2).

### Perceived stress

**Figure 3:**
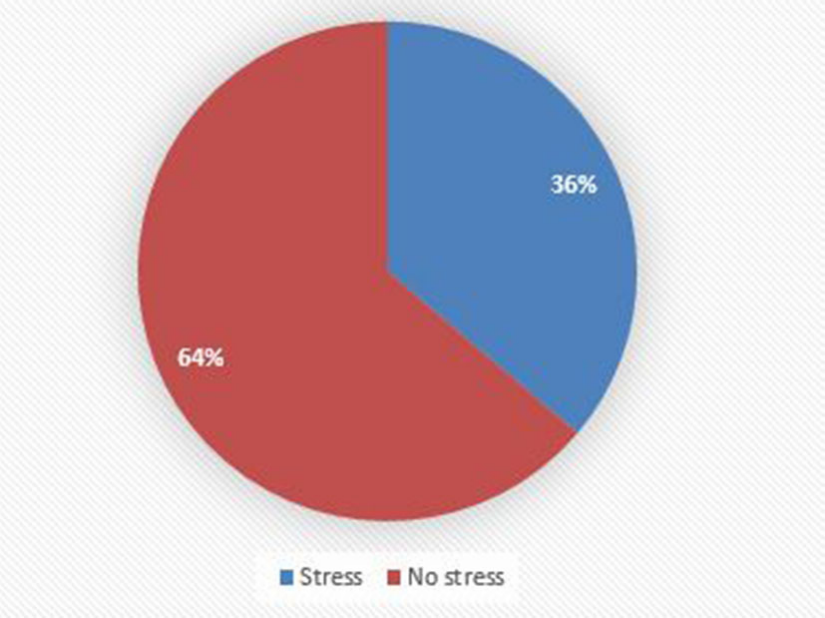
Psychological status of female college students, Debre Berhan, Ethiopia, 2021 (*n* = 395).

Out of the total respondents, 143 (36%) of college students had perceived stress (Figure 3).

### Substance use

Around 16 percent of the total respondents had consumed alcohol in the past. Almost all (98.4%) individuals who had a history of drinking alcohol had consumed alcohol in the previous 30 days. The types of alcohol they drank were beer 30 (49.2%), wine 12 (19.7%), or traditional drinks like local beer. Concerning the frequency of drinking alcohol, 10 (16.4%), 30 (49.2%), and 21 (34.4%) of the respondents drunk daily, weekly, and monthly respectively. In this study, none of the respondents had chewed chat or smoked cigarettes.

### Sleeping hours

Concerning the sleeping hours of the study respondents; 59 (14.9%), 212 (53.7%), and 124 (31.4%) of the respondents slept <5, 6–8, and 9 h and more per day, respectively.

### Medical and gynecological history

Of all study respondents, 385 (97.5%) had no history of gynecological problems. Of those who had a history of gynecological problems, 10 (2.5%) of the respondents had a history of sexually transmitted disease. Ninety-two of the study respondents (23.3%) started sexual activity and around 8% of them used emergency contraceptives. Regarding the medical history of the respondents, 2 (0.5%), 10 (2.5%), and 11 (2.8%) had a history of Diabetic Mellitus, goiter, and head injury, respectively.

### Patterns of the menstrual cycle

In this study, 132 (33.4%) [(95% CI 28.6–38.2)] of the respondents had an irregular menstrual cycle. Among those who had menstrual irregularity, 77 (58%) of the respondents had irregular onset and 16 (12.12%) of the respondents had heavy bleeding during menstruation. The mean age of menarche was 14.88 ± 1.419 years (Table 2).

**Table 2:** Patterns of the menstrual cycle among female college students, Debre Berhan, Ethiopia, 2021 (*n* = 395).

**Variable**	**Categories**	**Frequency (*n*)**	**Percent (%)**
Frequency of menstrual cycle	<21 days	20	5.1
	Between 21 and 35days/ normal	362	91.6
	>35 days/infrequent	13	3.3
Duration of menstrual flow	≤8 days/normal	382	96.7
	>8 days/prolonged	13	3.3
Perception on menstrual blood flow (volume)	2–4 pads/day (light)	327	82.8
	<2 pads/day (normal)	52	13.2
	>4 pads/day (heavy)	16	4.1
Inter menstrual bleeding	No	384	97.2
	Yes	11	2.8
Regularity of onset/intermenstrual difference	<10/regular	318	80.5
	≥10 days/irregular	77	19.5
Overall menstrual cycle	Regular	263	66.6
	Irregular	132	33.4

### Factors associated with menstrual irregularity

Bivariate analysis for each independent variable has been used to assess factors associated with menstrual irregularity, and then the age of respondent, body mass index, sleeping hour, age of menarche, psychological stress, residence, and head injury were statistically associated with menstrual cycle irregularity. Statistical significance was accepted at *p* < 0.25 (Table 3).

**Table 3:** Factors associated with menstrual irregularity of female college students, Debre Berhan, Ethiopia, 2021 (*n* = 395).

**Variables**	**Categories**	**Irregular Menstruatio**		**COR (95% of CI)**	**AOR (95% of CI)**
		**Yes**	**No**		
Age	≤20 years	110	117	4.94 (1.64–14.84)^*^	3.88 (1.25–12.18)^**^
	21–24 years	18	125	0.765 (0.233–2.455)	0.549 (0.159–1.900)
	≥25 years	4	21	1	1
Residence	Rural	88	122	2.311 (1.495–3.573) ^*^	1.485 (0.888–2.483)
	Urban	44	141	1	1
Age of menarche	≤ 12 years	13	5	5.57 (1.9–16.2)^*^	4 (1.2–13.9)^**^
	≥15 years	42	93	0.97 (0.62–1.52)	1.007 (0.59–1.71)
	13–14 years	77	163	1	1
Perceived stress	Have no stress	97	155	1	1
	Have stress	35	108	1.93 (1.22–3.05)^*^	2 (1.53–3.65)^**^
BMI	Underweight	34	43	1.07 (0.56–2.03)	0.995 (0.48–2.05)
	Overweight	30	33	2.5 (1.17–3.83)^*^	2.18 (1.13–3.23)^**^
	Normal	68	187	1	1
Sleeping hours	≤ 5 h	30	29	2.63 (1.38–5)^*^	2.26 (1.04–4.93)^**^
	≥9 h	35	89	1.18 (0.72–1.91)	1.365 (0.758–2.46)
	6–8 h	67	145	1	1

* P < 0.25;

** P < 0.05.

The above variables which showed a *p* < 0.25 during bivariate analysis were used in multivariate logistic regression to control confounders. Age of respondent, body mass index, sleeping hour, age of menarche, and psychological stress were the variables that showed significant association on multivariate logistic regression. The significant association was also declared at *p* < 0.05 (Table 3).

Students whose age was less than 20 years old were 3.88 times [AOR = 3.88, 95% CI (1.25–12.18)] more likely to experience irregular menstrual cycles than those aged 25 years and above. Those with menarche ≤12 years had 4 times greater [AOR = 4, 95% CI (1.18–13.9)] risk for menstrual irregularity than those who were seen with menses at 13–14 years old. Similarly, those respondents who were overweight were 2 times more likely [AOR =2.18, 95% CI (1.13–3.23)] to have more menstrual cycle irregularity as compared to those who were normal weight. On the other hand, participants who slept ≤ 5 h/days were 2.26 times [AOR = 2.26, 95 CI (1.04–4.93)] more likely to experience irregular menstrual cycles than those who were sleeping 6–8 h. per day. Finally, participants who had perceived stress were 2 times [AOR = 2, 95% CI (1.53–3.23)] more likely to have an irregular menstrual cycle than those who did not (Table 3).

## Discussion

This institution-based cross-sectional study was done on menstrual cycle irregularity and associated factors among college students in Debre Berhan town. The magnitude of menstrual cycle irregularity in the current study is 33.4% (132). This finding is consistent with previous studies conducted in Ethiopia, Japan, and Saudi Arabia (11, 25, 26). However, the finding of the current study is lower than studies conducted in Ethiopia Bahir Dar University, Sudan, Malaysia, and Lebanon (10, 27–29). On the other hand, the current study is higher than the study conducted in Adama, and India studies (30, 31). These differences could be attributable to the respondents' age ranges, stress levels, where they live, or undiscovered medical and gynecological issues.

Students whose age was less than 20 years old were shown to be 3.88 times more likely than the age group of ≥25 years to have irregular menstrual cycles in this study. This is in line with a study conducted in India (32). Menstrual irregularity is common in the first 2 years of menarche and is related to hypothalamus-pituitary-ovarian axis maturation. For the first few years after menstruation begins, long, irregular cycles are common. The irregularity during this time is due to anovulatory cycles. In youngsters, this often happens because their bodies have not yet settled into a pattern of regular menstrual cycles.

This study revealed that early menarche increases the risk of having irregular menstruation four-fold compared to those who started menses at 13–14 years old. This finding is consistent with previous findings in Ethiopia Adama, Lebanon, and Taiwanese college students (29, 30, 33). This might be because the girls at an early age might be exposed to stress, and the part of the brain that regulates periods is influenced by stress. Furthermore, at a young age, the hypothalamic-pituitary-ovarian axis is not mature enough to regulate the release of steroid hormones that are critical for controlling the menstrual cycle.

Our studies show overweight students were at two-fold greater risk for menstrual irregularity compared to those who were of normal weight. This result is consistent with other studies conducted in Ethiopia Adama, Lebanon, Taiwan, and India (11, 29, 30, 33, 34). This could be due to hormonal abnormalities caused by obesity and being overweight (body fat). Being overweight or obese have been linked to increased testosterone, disrupted estrogen, and decreased sex hormone-binding globulin (SHBG). Higher levels of testosterone have been associated with polycystic ovary syndrome, which is related to ovulatory dysfunction and menstrual irregularity. Clinical studies have also shown elevated total and free androgen levels and depressed SHBG in overweight/obese women with amenorrhea or oligomenorrhea (35–37). D-chiro-inositol downregulates the expression of aromatase, reducing the conversion of androgens into estrogens. Myo-insitol is currently used to treat menstrual irregularity in polycystic ovary syndrome (PCOS) (38).

Perceived stress made menstrual irregularities 2 times more likely. This finding is in line with studies conducted in Adama, Saudi Arabia, and India (26, 30, 31). This could be due to the psychological stress that might affect the coordination of HPO-Axis. The hypothalamus, which controls the pituitary gland, the body's master gland, which, in turn, controls the thyroid, adrenal glands, and the ovaries, which all work together to maintain hormones, is clearly affected by stress. This incoordination leads to irregular menstruation.

Our findings show those respondents who sleep < 5 h were two times more at risk of having menstrual irregularity than those who sleep 6–8 h per day. This result is consistent with studies done in Ethiopia (Adama and Debre Berhan University), China, and Korea (11, 13, 30, 39). This could be related to sleep disruption, which can cause hormone imbalances, which can disrupt the menstrual cycle, and cause discomfort and stress. The hypothalamic-pituitary-adrenal axis is crucial for maintaining alertness and sleep regulation. Dysfunction of this axis at any level can disrupt sleep. The hypothalamic-pituitary- axis activities regulate the menstrual cycle.

### Limitation of the study

Our study was carried out during the global pandemic of COVID-19, which might have increased the students' level of stress, which is one of the strong risk factors for menstrual irregularities. This study was also conducted in a single study area.

## Conclusion

In this study, more than one-third of the college girls in Debre Berhan town were shown to have experienced menstrual irregularity. Being less than 20 years old, having a history of early menarche, being overweight, and perceived stress were variables significantly associated with menstrual irregularity.

## Recommendations

There is a need to plan strategies for girls to help them control menstrual irregularity; girls should control their weight and lead a healthy lifestyle, including getting adequate sleep. In addition, it is better to give training for students in life skills like study and time management skills to avoid or minimize their stress and to help them achieve adequate rest or sleep. Girls who follow healthy living choices may be able to control menstrual irregularity and lessen its impact on everyday activities as well as academic performance.

## Data availability statement

The original contributions presented in the study are included in the article/supplementary material, further inquiries can be directed to the corresponding author/s.

## Ethics statement

The Ethical Review Board of Debre Berhan University's College of Health Sciences provided ethical approval. Formal letters acquired from the Amhara Regional Health and Teaching Bureau were used to contact with the Dean and Heads of Department. Each study respondent gave written consent after being informed of the study's purpose and/or objectives.

## Author contributions

YM considered the study, analyzed the data, and wrote the manuscript. HM, GWo, MT, and GWa were involved in the interpretation of the data and contributed to manuscript preparation. All authors read and approved the final manuscript.

## Conflict of interest

The authors declare that the research was conducted in the absence of any commercial or financial relationships that could be construed as a potential conflict of interest.

## Publisher's note

All claims expressed in this article are solely those of the authors and do not necessarily represent those of their affiliated organizations, or those of the publisher, the editors and the reviewers. Any product that may be evaluated in this article, or claim that may be made by its manufacturer, is not guaranteed or endorsed by the publisher.

## Abbreviations

AUB: Abnormal Uterine Bleeding
AOR: Adjusted odds ratio
BMI: Body Mass Index
CI: Confidence Interval
COR: Crude Odds Ration
HPO: Hypothalamic Pituitary Ovary
PSS: Perceived Stress Scale
SHBG: Sex Hormone-Binding Globulin.
